# Feasibility and initial experience of assessment of mechanical dyssynchrony using cardiovascular magnetic resonance and semi-automatic border detection

**DOI:** 10.1186/1532-429X-10-49

**Published:** 2008-11-04

**Authors:** Ralf Koos, Mirja Neizel, Georg Schummers, Gabriele A Krombach, Sven Stanzel, Rolf W Günther, Malte Kelm, Harald P Kühl

**Affiliations:** 1 Department of Cardiology, University Hospital RWTH Aachen, RWTH Aachen University, Pauwelsstrasse 30, 52057 Aachen, Germany; 2 TomTec Imaging, Unterschleissheim, Germany; 3 Department of Diagnostic Radiology, University Hospital RWTH Aachen, RWTH Aachen University, Pauwelsstrasse 30, 52057 Aachen, Germany; 4 Institute of Medical Statistics, University Hospital RWTH Aachen, RWTH Aachen University, Pauwelsstrasse 30, 52057 Aachen, Germany

## Abstract

**Background:**

The systolic dyssynchrony index (SDI) has been introduced as a measure of mechanical dyssynchrony using three-dimensional echocardiography to select patients who may benefit from cardiac resynchronization therapy (CRT). However, three-dimensional echocardiography may be inadequate in a number of patients with suboptimal acoustic window and no single echocardiographic measure of dyssynchrony has proven to be of value in selecting patients for CRT. Thus, the aim of this study was to determine the value of cardiovascular magnetic resonance (CMR) for the assessment of the SDI in patients with reduced LV function as well as in healthy controls using semi-automatic border tracking.

**Methods:**

We investigated a total of 45 patients including 35 patients (65 ± 8 years) with reduced LV function (EF 30 ± 11%) and a wide QRS complex as well as 10 control subjects (42 ± 21 years, EF 70 ± 11%). For cine imaging a standard SSFP imaging sequence was used with a temporal resolution of 40 frames per RR-interval. Quantitative analysis was performed off-line using a software prototype for semi-automatic border detection. Global volumes, ejection fraction and the SDI were calculated in each subject. SDI was compared with standard echocardiographic parameters of dyssynchrony.

**Results:**

The mean SDI differed significantly between patients (14 ± 5%) and controls (5 ± 2%, p < 0.001). An exponential correlation between the EF and the SDI was observed (r = -0.84; p < 0.001). In addition, a significant association between the SDI and the standard deviation of time to peak systolic motion of 12 LV segments (Ts-SD) determined by echocardiography was observed (r = 0.66, p = 0.002).

**Conclusion:**

The results of this preliminary study suggest that CMR with semi-automatic border detection may be useful for the assessment of mechanical dyssynchrony in patients with reduced LV function.

No trial registration due to recruitment period between October 2004 and November 2006

## Background

Several studies have shown that the presence of intra-left ventricular (LV) dyssynchrony is one of the main predictors of response to cardiac resynchronization therapy (CRT) [1-3]. The surface electrocardiogram may not be optimal for patient selection because electrical dyssynchrony (wide QRS complex) does not accurately reflect intra-LV dyssynchrony [4,5]. Tissue Doppler imaging (TDI) can assess LV dyssynchrony, i.e. the mechanical delay between septum and lateral wall contraction [2,3,6,7]. In single center studies TDI as well as strain and strain rate imaging have been shown to be potentially useful in the selection of candidates for CRT [2,3,6-8]. However, these techniques have important technical limitations [9]. In case of TDI regional systolic velocities may reflect passive motion of segments due to heart motion or tethering by adjacent segments. In addition, it is difficult to measure myocardial velocities in the apical segments of the LV.

More recently volumetric real time three-dimensional echocardiography has been used in conjunction with semi-automatic border detection algorithms to evaluate heart-failure patients for the presence of mechanical asynchrony [10,11]. Myocardial wall motion has been shown to be accurately quantified by volume-time curve analysis with 3D analysis software [11]. The systolic dyssynchrony index (SDI) defined as the standard deviation of the time to minimal systolic volume of all 16 segments was introduced as a measure of mechanical dyssynchrony [10]. This index has been shown to be potentially useful for selection of patients who may benefit from CRT [10]. However, three dimensional echocardiography may be inadequate in a number of patients due to limited temporal resolution and suboptimal acoustic window. In addition, no single echocardiographic measure of dyssynchrony has proved to be of value in selecting patients for CRT [12] and has been validated against mortality. Therefore, novel techniques to quantify mechanical dyssynchrony using cardiovascular magnetic resonance (CMR) may be of potential value.

Thus, the primary aim of this study was to compare the SDI in patients with reduced LV function and in healthy controls using cine CMR and semi-automatic border detection algorithms.

## Methods

### Patients and controls

The study group of this feasibility study consisted of 45 subjects including 35 heart failure (HF) patients (65 ± 8 years, 22 men) with reduced systolic LV function (EF 30 ± 11%) and wide QRS complex ≥ 120 ms (mean QRS duration 158 ± 22 ms). Ten normal subjects without cardiac disease (42 ± 21 years, EF 70 ± 11%) with normal QRS duration (83 ± 5 ms on electrocardiogram) were evaluated for comparison. The inclusion criteria for the HF patients were as follows: 1) heart failure New York Heart Association (NYHA) class III or IV 2) known ischemic (n = 13) or non-ischemic cardiomyopathy (n = 22) with at least a moderately impaired LV function (EF ≤ 40%) as assessed by CMR and 3) wide QRS complex ≥ 120 ms with left bundle branch block or interventricular conduction delay. Patients with acute coronary syndromes were excluded from the study. The patients were recruited between October 2004 and November 2006.

The ischemic or non-ischemic etiology of the cardiomyopathy was assessed using invasive cardiac catheterization. We performed cine CMR in all patients and control subjects. In addition, we retrospectively analysed echocardiographic data of the HF patients.

Informed consent was obtained from each patient prior to the investigation in accordance with local Ethics Committee requirements.

### CMR protocol and generation of volume-time curves

Cine CMR was performed on a 1.5 T whole-body MR scanner (Intera, Philips Medical Systems, Best, The Netherlands) with electrocardiographic gating. Images were obtained using a five element cardiac synergy coil during repeated breath-holds (approximately 7 to 15 seconds). For cine imaging a standard SSFP imaging sequence (imaging parameters: echo time (TE) 1.8 ms, repetition time (TR) 3.6 ms, spatial resolution 1.8 × 1.5 mm^2^, slice thickness 8 mm) was used with a temporal resolution of 40 frames per RR-interval. Three long-axis images and a stack of 10 to 12 short axis images covering the complete LV were acquired.

**Figure 1:**
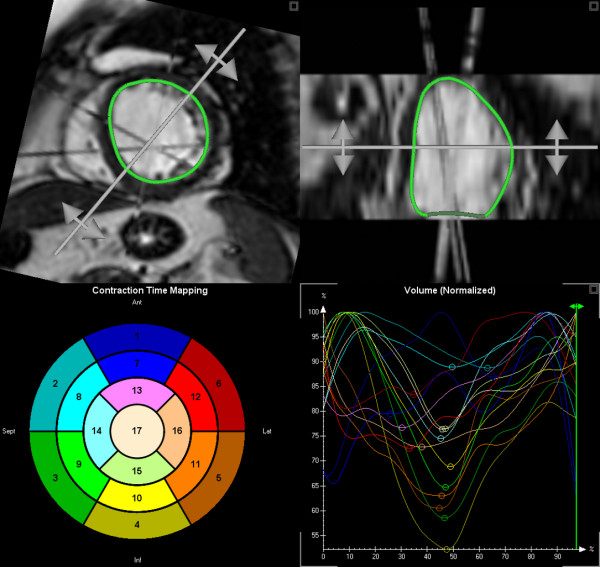
Semi-automatically segmented left ventricle (upper panels) with plot of segmental volume-time curves (right lower panel) and bulls eye plot of the 17 segment model (left lower panel).

The data set was transferred to a separate workstation and reconstructed off-line to a 4D volume data set. Quantitative analysis was performed using a software prototype for semi-automatic border detection (TomTec, Unterschleissheim, Germany). Endocardial borders were manually outlined in the three long-axis images at end-diastole and end-systole. Based on these initial contours LV casts are automatically calculated for each of the 40 time frames per RR interval providing a global volume-time-curve. Manual editing of endocardial borders in selected short-axis slices was possible. These changes in 2D images were subsequently adopted in the 3D data set. The cast were automatically subdivided into 17 subsegments resulting in 17 segmental volume-time curves (Fig. 1). The SDI was calculated from the time taken to reach minimum regional systolic volume for each segment as a percentage of the cardiac cycle. The SDI is defined as the standard deviation of these timings [10]. Higher SDI denotes increasing intraventricular dyssynchrony. In addition, global volumes and ejection fraction were calculated in each patient.

### Echocardiography

We retrospectively analysed echocardiographic data off-line using commercial software (Echopac 6.1, General Electric-Vingmed) in 32 of 35 (91%) HF patients. These patients received a comprehensive echocardiographic study using a commercially available ultrasonographic system (GE Vingmed, Vivid 7, Horten, Norway). Interventricular mechanical dyssynchrony was assessed by determination of aortic and pulmonary pre-ejection times. A difference of more than 40 ms between the pulmonary and the aortic pre-ejection times (dPEP) was considered as significant delay [13]. For assessment of intraventricular mechanical asynchrony, measurement of septal to posterior wall thickening delay (SPWMD) by M-mode echocardiography was performed using a cut-off of ≥ 130 ms indicating asynchrony [14]. In addition, in 20 patients (57%) tissue Doppler images from three apical views (2, 3 and 4 chamber views) were available and the standard deviation of the time to peak systolic motion in 12 segments (Ts-SD) according to Yu et al was determined [1]. A cut-off of Ts-SD = 34.4 ms was considered as measure for the presence of asynchrony [1].

### Statistics

Continuous variables are expressed as mean values ± standard deviation. Categorical data are presented as frequencies for the study population as well as for the control group and were compared using the Chi-Square test. The unpaired Student's t test was used for comparisons of continuous variables, i.e. for comparison of the ejection fraction, LV volume and SDI between patients and control subjects. The degree of association between SDI and EF as well as between SDI and echocardiographic parameters of asynchrony (Ts-SD, SPWMD, dPEP) was investigated by computing the Pearson's correlation coefficient. In addition, intraobserver and interobserver correlations were evaluated by using the Pearson's correlation coefficient.

Intraobserver variability of SDI was derived from manually outlined endocardial borders in the three long-axis images at end-diastole and end-systole from CMR of 10 randomly selected patients. Studies were performed by the same observer on 2 occasions 3 weeks apart. Interobserver variability was derived from the same subjects, performed by two blinded observers. Both intra- and interobserver variabilities were calculated as the standard deviation of the absolute difference between 2 measurements divided by the mean of both measurements, and expressed as a percentage. In Bland Altman analyses, the intraobserver and interobserver agreements, expressed in terms of the mean difference ± 2 standard deviation were evaluated.

The global significance level for all statistical tests conducted was chosen to α = 0.05. Statistical analysis was performed with the use of SPSS 10.0 (SPSS Inc., Chicago, USA).

## Results

### Clinical characteristics and CMR results of patients and controls

The clinical baseline characteristics of the patient population with heart failure and the healthy control subjects are demonstrated in Table 1.

**Table 1:** Clinical characteristics of the study cohort and controls

	Patients with HF (n = 35)	Controls (n = 10)
Men	22 (63%)	7 (70%)
Ischemic cardiomyopathy	13 (37%)	0
Non-ischemic cardiomyopathy	22 (63%)	0
New York Heart Association		
class II/III/IV	6/25/4	0/0/0
Hypertension	19 (54%)	1 (10%)
Diabetes mellitus	5 (14%)	0
Hypercholesterolemia	21 (60%)	2 (20%)
Smoker	22 (63%)	3 (33%)
QRS duration (ms)	158 ± 22	83 ± 5

Values are presented as mean ± S.D. or absolute values with percentage in parenthesis as appropriate.

**Figure 2:**
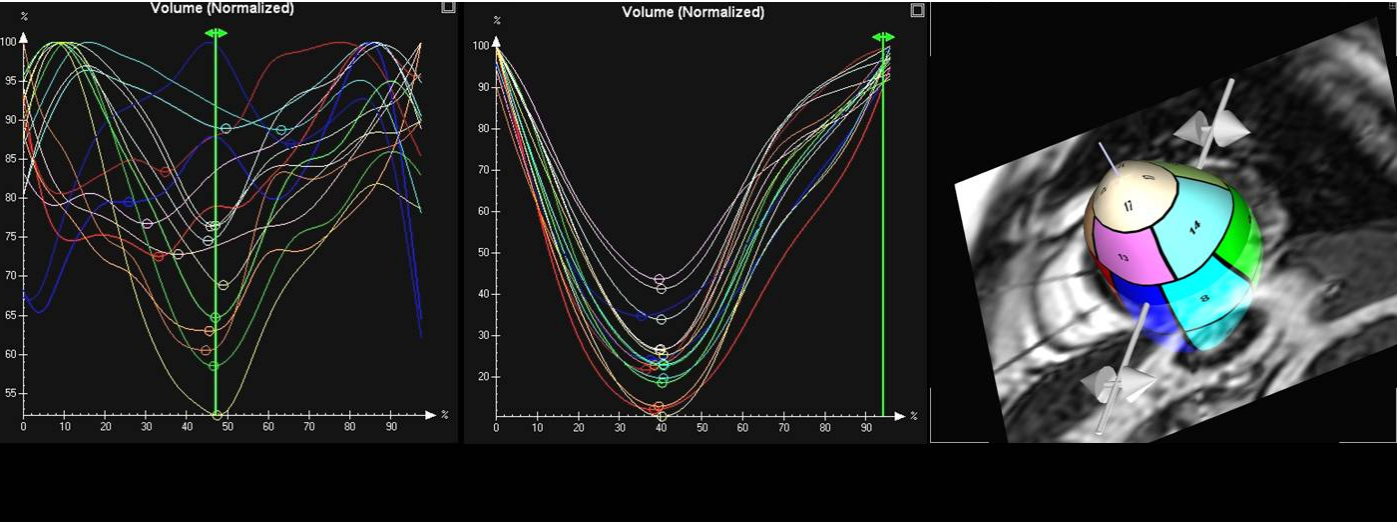
Segmental volume-time-curves for a patient with marked dyssynchrony (SDI 11%, left panel) and a normal control subject (SDI 3%, mid panel); right panel: view on 3D LV cast.

**Figure 3:**
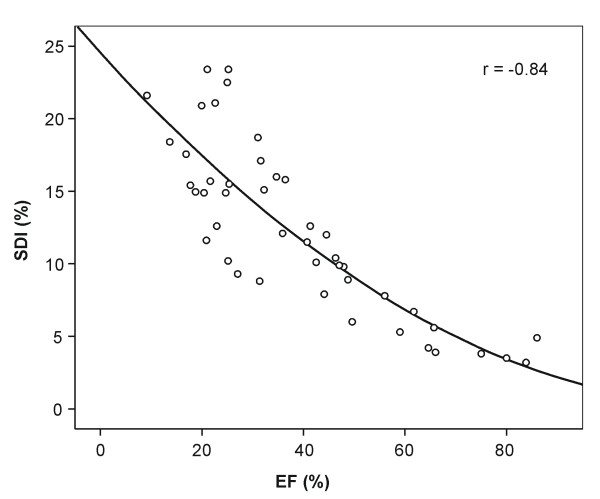
Graph shows significant correlation between EF and SDI (r = -0.84, p < 0.001).

Data analysis using the semi-automatic software was feasible in all patients. Manual correction of endocardial contours had to be performed in most of the patients (n = 39, 87%). The mean time required for complete analysis was 10 minutes. However, major corrections defined by an additional time required for manual editing of endocardial borders in selected short-axis slices > 5 minutes (range 6–15 minutes) were needed in only a smaller proportion of patients (n = 10, 22%). Eight (80%) of these patients belonged to the HF group. In patients with HF the EF was significantly lower (EF 30 ± 11%) compared to the control subjects (EF 70 ± 11%, p < 0.001). In addition, LVEDV and LVESV measured by CMR were larger in HF patients than in controls (Table 2). The mean SDI was significantly increased in patients with HF (14 ± 5%, range 6.0%–23.4%) compared to control subjects (4.9 ± 1.5%, range 3.2%–7.8%; p < 0.001). An example of segmental volume-time-curves for a patient with marked dyssynchrony and a normal control subject is shown in Fig. 2. A significant exponential correlation between the mean SDI and EF was observed (r = -0.84, p < 0.001; Fig. 3). The correlation between mean SDI and EF for patients with ischemic cardiomyopathy and non-ischemic cardiomyopathy was r = -0.59 and r = -0.76, respectively. In addition, a strong correlation between the QRS complex duration and SDI was noted (r = 0.67, p < 0.001).

**Table 2:** CMR results of volumes and ejection fraction

Parameters	Heart failure patients (n = 35)	Controls (n = 10)	p value
LVEDV (mL)	312 ± 122	145 ± 46	<0.001
LVESV (mL)	225 ± 118	48 ± 27	<0.001
EF (%)	30 ± 11	70 ± 11	<0.001
SDI (%)	14.5 ± 4.7	4.9 ± 1.5	<0.001

Values are presented as mean ± S.D.

LVEDV = left ventricular end diastolic volume, LVESV = left ventricular end systolic. volume, EF = ejection fraction, SDI = systolic dyssynchrony index.

Intraobserver and interobserver variability for SDI was 2.2% and 3.4%, respectively. In addition, intraobserver and interobserver correlation was r = 0.99 and r = 0.94, respectively. In Bland Altman analyses, the intraobserver and interobserver agreements, expressed in terms of the mean difference ± 2 standard deviation (upper and lower limits of agreement) were 0.02 (-1.87 to 1,91%) and 0.16 (-3.45 to 3.77%), respectively.

### Echocardiography

**Figure 4:**
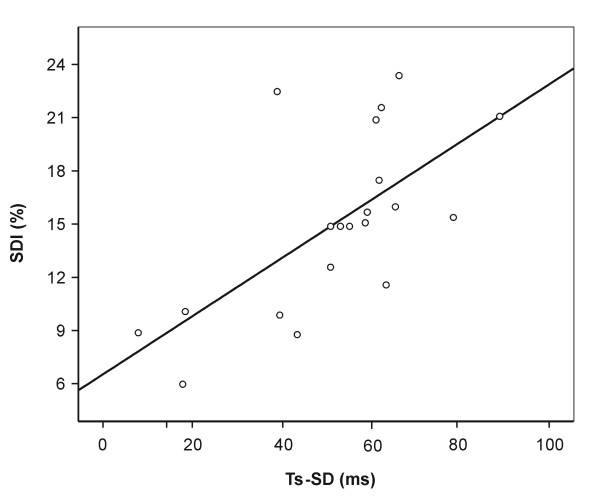
Graph shows linear association between SDI and echocardiographically determined Ts-SD (r = 0.66, p = 0.002).

A significant association between the SDI assessed by CMR and the standard deviation of time to peak systolic motion of 12 LV segments (Ts-SD) determined by Tissue Doppler imaging was observed (r = 0.66, p = 0.002, n = 20, Fig. 4). However, no significant associations between the SDI and SPWMD (r = 0.31, p = 0.11, n = 29) as well as between the SDI and dPEP (r = 0.04, p = 0.82, n = 32) were noted.

## Discussion

The results of this preliminary study show that myocardial wall motion can be quantified by volume-time curve analysis for assessment of mechanical dyssynchrony using CMR with semi-automatic border detection in analogy to 3D echocardiography. Using 3D imaging techniques it is possible to integrate the function of all 17 segments into the SDI which can be used to quantify the severity of LV dyssynchrony.

There is currently no gold-standard for the assessment of LV dyssynchrony. As electrical dyssynchrony does not accurately reflect intra-LV dyssynchrony, QRS-duration was found to be a poor predictor of CRT long term response [7,15]. Ventricular mechanical dyssynchrony can be demonstrated by numerous echocardiographic parameters. However, these techniques have important technical limitations [9]. In case of SPWMD, this parameter may not be useful in many patients, especially those with ischemic cardiomyopathy, in whom the inter-ventricular septum may be akinetic. This may partially contribute to the worse association between SDI and SPWMD observed in this study. The lack of correlation between SDI and dPEP may at least in part result from the fact that dPEP is a measure of interventricular dyssynchrony whereas the SDI reflect intra-LV dyssynchrony.

Real time 3D echocardiography has been shown to provide a precise evaluation of LV volumes even in conditions of altered load [16]. Kapetanakis et al [10] used 3D echocardiography for guiding CRT and introduced the concept of the SDI. Despite the advantages of 3D echocardiography for evaluation of LV asynchrony, there are also limitations. LV volume analysis is image sector and quality dependent i.e. clear endocardial borders and complete LV cavity acquisition is required, which may be difficult in severely dilated left ventricles. Moreover, 3D echocardiography has a relatively low temporal resolution compared to other echo modalities such as M-Mode or TDI.

As no single echocardiographic measure of dyssynchrony has proven to be of value in selecting patients for CRT [12], CMR may be an alternative approach to select patients for CRT. In addition, late gadolinium enhancement (LGE) is able to determine the extent and transmurality of scar tissue. Recent data show that the total scar burden is an important factor influencing response to CRT [17]. Thus, evaluation for viability, scar tissue and mechanical dyssynchrony at the same time using CMR may be important in the selection process for CRT candidates. Moreover, CMR is a powerful imaging modality for quantitative assessment of 3D myocardial structure und function. Measurements of end diastolic, end systolic volumes as well as ejection fraction are accurate and highly reproducible [18]. A quantitative analysis of regional LV function has been performed by MR tagging and strain encoded imaging [19]. However, the length of the post processing analysis procedure may often pose limitations to the clinical application.

In this CMR study a semi-automatic border detection prototype for quantitative analysis of LV dyssynchrony was used. The SDI values for normal subjects as well as for patients with HF in this study are in excellent agreement to previously published data using real-time 3D echocardiography [10]. In addition, in HF patients an association between the SDI and the "echocardiographic dyssynchrony index", the Ts-SD, was shown in our study. Moreover, our study confirms the findings of Kapetanakis et al [10] that mechanical dyssynchrony is increasingly prevalent with worsening LV function which is supported by the reported correlation between the SDI and the EF. As 3D imaging not only allows to accurately evaluate EF but also demonstrates changes in regional myocardial motion by calculating regional volume-time curves, this may be important for preoperative planning before CRT. A recent CMR study evaluated a tissue synchronization index showing CMR to be a powerful method for assessing LV dyssynchrony and also for predicting mortality after CRT [20]. In contrast to this study using semi-automatic border tracking, left ventricular volumes were quantified using manual planimetry of all short axis cine images in the former study [20]. As manual planimetry of short axis images is possibly afflicted with inaccuracies and is more time consuming this poses limitations to its application in clinical routine. The ability of 3D imaging with CMR to discriminate between patients with heart failure and healthy control patients, to measure EF accurately, to demonstrate scar tissue and to assess SDI with high reproducibility from a 3D data set reflects its capability to image the entire left ventricle with superior spatial resolution. Thus, CMR may be a powerful method in the selection process for CRT candidates with the ability to identify LV dyssynchrony and to demonstrate scar tissue, which may prohibit response to CRT, at the same time.

## Limitations

The study population was relatively small. In addition, we did not assess a potential influence of infarct related wall thinning or motion abnormality on the calculation of the SDI. Thus, further prospective studies in larger populations also in comparisons to other imaging modalities are required to confirm this methodology to assess LV dyssynchrony and to compare with patient outcome.

## Conclusion

The results of this feasibility study suggest that CMR with semi-automatic border detection may be useful for the assessment of mechanical dyssynchrony in patients with reduced LV function.

## Competing interests

The authors declare that they have no competing interests.

## Authors' contributions

RK conceived and designed the study, analyzed the data and drafted the manuscript. MN operated the CMR scanner and helped draft the manuscript. GS stored the data and participated in analyzing the CMR data. GAK operated the CMR scanner and helped draft the manuscript. SS performed the statistical analysis. RWG and MK participated in study design, coordination and scientific input. HPK participated in analysing the CMR data as well as in scientific input. All authors read and approved the final manuscript.
